# Usnic Acid and Its Topical Use—A Concise Review

**DOI:** 10.3390/molecules31122183

**Published:** 2026-06-22

**Authors:** Gabriela Siedlarczyk, Irma Podolak, Agnieszka Galanty

**Affiliations:** 1 Doctoral School of Medical and Health Sciences, Jagiellonian University Medical College, 16 Łazarza Str., 31-530 Cracow, Poland; gabriela.siedlarczyk@doctoral.uj.edu.pl; 2 Department of Pharmacognosy, Jagiellonian University Medical College, Medyczna 9, 30-688 Cracow, Poland; irma.podolak@uj.edu.pl

**Keywords:** usnic acid, lichen extracts, melanoma, wound healing, liposomes, topical

## Abstract

Usnic acid (UA), a prominent lichen secondary metabolite, exhibits a unique dual therapeutic profile in dermatology, though its clinical translation is limited by systemic hepatotoxicity and poor solubility. This review comprehensively evaluates the topical efficacy, molecular mechanisms, and advanced formulation strategies of UA enantiomers and UA-rich extracts. A literature search across PubMed, Scopus, and Google Scholar identified 36 original publications focusing on anti-melanoma activity, photoprotection, and tissue regeneration. In vitro studies demonstrate that UA induces apoptosis in resistant melanoma cell lines (A375, HTB-140) via extrinsic/intrinsic pathways, with (−)-UA effectively overcoming doxorubicin resistance. Conversely, in non-cancerous models, low concentrations of UA accelerate wound and burn healing by upregulating vascular endothelial growth factor (VEGF), stimulating fibroblast proliferation, and optimizing extracellular matrix remodeling while preventing hypertrophic scarring. To mitigate skin sensitization and systemic risks, advanced drug delivery systems—including liposomes, nanoemulsions, chitosan nanogels, and electrospun scaffolds—have been developed, significantly enhancing skin permeability and localized dermal retention. Ultimately, the development of bio-functionalized smart dressings and targeted nano-formulations represents the most viable path toward unlocking the full clinical potential of UA in modern dermatological and oncological care.

## 1. Introduction

**Figure 1:**
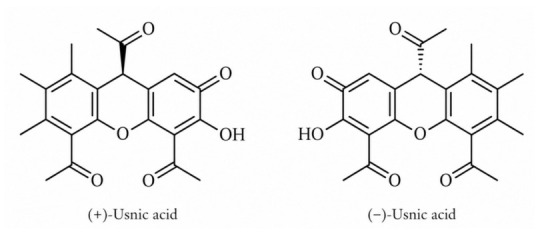
Chemical structures of usnic acid (UA) enantiomers: (+)-usnic acid and (−)-usnic acid.

Lichens (Latin *Lichenes*) or lichenized fungi are symbiotic organisms composed of an algal and/or a cyanobacterial and a fungal unit, accompanied by some bacterial communities [1]. Such coexistence results in the production of structurally unique and potentially biologically active compounds, among which the secondary metabolite UA remains one of the most intriguing. It was first isolated in 1844 by the German scientist Knop from some *Usnea* species, after which the compound was named. Usnic acid exists as a chiral molecule in two enantiomers (Figure 1): dextrorotatory (+)-UA and levorotatory (−)-UA [2]. UA is widely distributed across various lichen genera, including *Usnea*, *Parmelia*, *Lecanora*, *Cladonia*, *Ramalina*, *Alectoria*, and *Evernia*, while some genera did not produce the compound [3]. The compound is of lipophilic nature, which results in its poor water solubility; thus, it is most often extracted from the lichen matrix with chloroform, ethyl acetate, or acetone [4].

The UA enantiomers exhibit a broad spectrum of biological activities, encompassing cytotoxic, antimicrobial, anti-inflammatory, and photoprotective properties [3]. Despite the extensive documentation of these biological traits, comparative data regarding the specific activities of the two enantiomers remain remarkably limited. The vast majority of published studies focus predominantly on (+)-UA, leaving a substantial gap in the understanding of (−)-UA. Furthermore, very few experimental designs evaluate both enantiomers simultaneously within the same study [5].

Crucially, the internal therapeutic application of UA is strictly limited by its documented systemic hepatotoxicity, resulting probably from the uncoupling of oxidative phosphorylation in mitochondria [6], which was recently reviewed in detail by Chen et al. [7]. Consequently, its biological potential should be shifted toward topical administration [8]. However, dermatological use also presents challenges, as UA is known to induce allergic contact dermatitis. Forestry workers and consumers using cosmetics containing this compound are the groups with the highest risk of sensitization [8,9,10]. Allergenic potential of UA typically manifests as eczematous lesions on the hands and face. Interestingly, significant immunological differences exist between the two isomers, with the dextrorotatory form (+)-UA demonstrating noticeably stronger allergenic potency [11,12]. However, it should be underlined that the reports on the allergic potential of UA are based on case studies and appear to be rare. Thus, despite these allergenic reports, UA continues to be utilized in commercial formulations, such as antiperspirants, highlighting the need for rigorous dermatological safety evaluations to fully unlock its safe therapeutic application [8]. In our previous review, we presented the anti-inflammatory properties of UA, demonstrating its interesting impact on a wide panel of inflammatory biomarkers, including nitric oxide (NO), tumor necrosis factor (TNF-α), and interleukin 6 (IL-6), supported by a number of in vitro and animal studies [11]. Of special attention are the anti-inflammatory effects reported for the skin inflammation in rats, where UA treatment significantly improved collagen quality and density, but also in granulation tissue and scar repair, comparable to sulfadiazine silver ointment used as the reference compound [13]. Thus, the aim of this review was to gather the published data to date on the impact of UA on different aspects of skin functioning and evaluate the results in terms of the potential future topical application of UA.

## 2. Literature Search Strategy

A comprehensive literature search was conducted across the PubMed, Scopus, and Google Scholar databases, covering all articles found up to January 2026. The search strategy involved combinations of keywords and logical operators, specifically: “usnic acid AND melanoma”, “usnic acid AND wound healing”, “usnic acid AND topical formulations”, and “lichen extract AND skin cells”. An additional criterion was introduced to include only English-language results.

In the first stage, a total of 428 articles were identified. After a thorough evaluation of the titles and abstracts, 245 items were excluded due to being duplicates, unrelated to dermatological applications, or focusing on non-cutaneous cancers. The remaining 183 articles underwent full-text evaluation based on strict inclusion and exclusion criteria. The inclusion criteria comprised original studies describing the effects of pure UA enantiomers or UA-rich lichen extracts on normal and cancerous skin cells, tissue regeneration, photoprotection, and advanced topical drug delivery systems (e.g., liposomes, nanogels). Review articles, in silico studies without experimental validation, and papers focused exclusively on systemic oral administration or general systemic toxicity were excluded.

**Figure 2:**
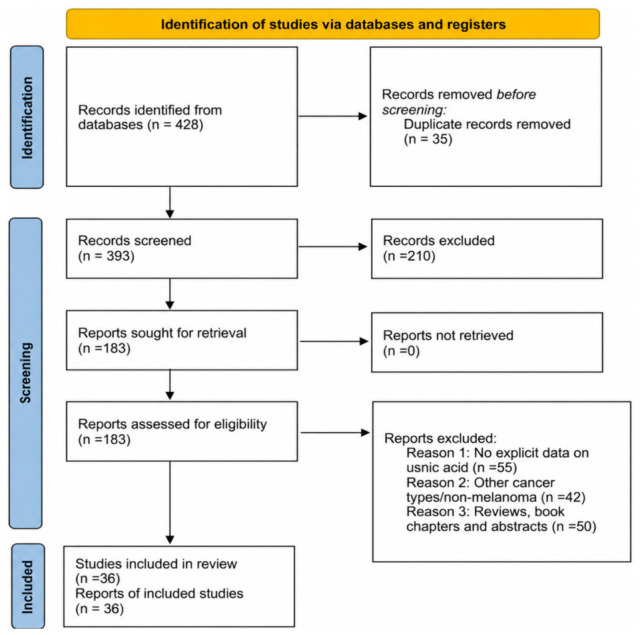
Diagram presenting the literature search and selection process for studies on usnic acid.

Following this manual selection process, 146 articles were excluded for not meeting the core criteria or lacking precise reference controls. Ultimately, 36 original publications directly relevant to the dual therapeutic profile of UA were selected and analyzed in this review (Figure 2).

## 3. Results

### 3.1. Impact of Usnic Acid on Normal and Cancer Skin Cells

**Figure 3:**
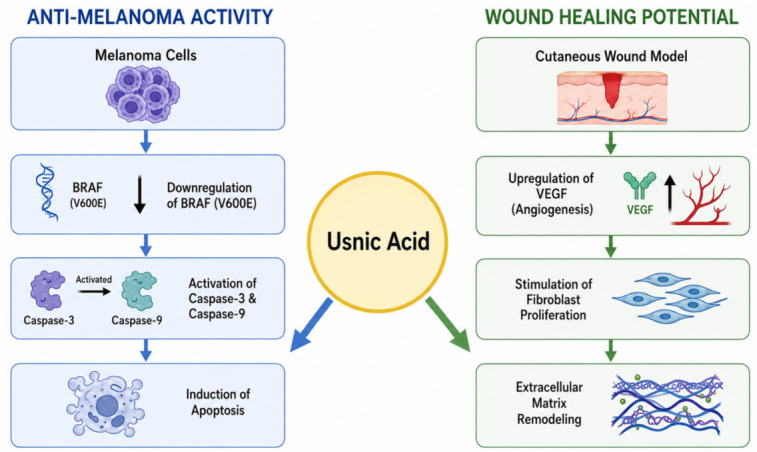
Dual therapeutic mechanisms of UA in dermatological applications. The left pathway illustrates the anti-melanoma activity via downregulation of the mutated BRAF (V600E) gene and subsequent activation of apoptotic Caspase-3 and Caspase-9. The right pathway outlines the wound healing potential through VEGF-mediated angiogenesis, fibroblast proliferation, and extracellular matrix remodeling. See Abbreviations section for details.

Most of the so-far existing studies described the impact of UA on skin cancer cells, mainly melanoma, while only a few studies focused on the effect on normal skin cells. In some studies on melanoma cells (Figure 3), the effect on normal cells was also described to verify the selectivity of cytotoxic potential. The details of the studies are presented in Table 1. It should be noted that the majority of the studies were performed for (+)-UA, while only one experiment compared the effectiveness of both enantiomers.

Two studies reported the inhibitory effect of UA on the proliferation of non-cancerous human HaCaT keratinocytes, as a simplified model of psoriasis in vitro. The results of the studies indicated a strong or moderate effect on keratinocyte proliferation, with an IC_50_ value of 2.1 µM (0.7 µM for the reference substance, anthralin), noted for the lactate dehydrogenase release assay [14] and EC_50_ values of 35 and 76 µM for the crystal violet and neutral red uptake assays, respectively [15].

Within the studies describing the impact of UA on skin cancer cells, the majority of the results concern human melanoma cell lines, while a few focused alsoalso focused on murine melanoma cells. In the experiment by Brandão et al., the cytotoxic potential of UA against human UACC-62 and murine B16-F10 melanoma cell lines and normal murine 3T3 fibroblasts was analyzed. The compound inhibited the growth of the melanoma cells, with GI_50_ values of 91.5 µM and 137.7 µM for UACC-62 and B16-F10 cells, respectively, which, compared to the reference doxorubicin (0.81 µM and 0.43 µM), indicates moderate activity of the tested compound against these tumor lines. In turn, the LC_50_ values for UA were 534.4 µM for the UACC-62 and >726.1 µM for the B16-F10 cells. This suggests the overall low sensitivity of both cell lines to UA. At the same time, UA revealed only limited toxicity to normal 3T3 fibroblasts [16]. Another study assessed the effect of UA on the viability, proliferation, apoptosis, motile activity, and actin cytoskeleton organization of HTB-140 human melanoma cancer cells, in comparison with normal human skin fibroblasts (HSF) [17]. The antiproliferative effect of UA on HTB-140 cells was moderate and time-dependent (Table 1). In addition, UA exhibited only a slight pro-apoptotic effect on HTB-140 cells, causing a limited increase in the number of apoptotic cells. Moreover, UA exerted a pronounced anti-invasive effect on melanoma cells, particularly by inhibiting cell motility and disrupting actin cytoskeleton organization. At a concentration of 10 μg/mL, the trajectory length of HTB-140 cells decreased by approximately 55%, while total cell displacement was reduced by about 70% compared to the control. After 24 h of incubation, the cells almost completely lost their ability to migrate. The effect also manifested in significant rearrangements of the actin cytoskeleton, including disorganization of actin filaments and a reduction in focal adhesion structures. Importantly, normal skin fibroblasts were significantly less sensitive to the compound, and cytostatic effects were observed only after prolonged incubation times [17]. In their subsequent study, the same authors compared the effects of UA enantiomers on three human melanoma cell lines: HTB-140, A375, and WM793, differing in metastatic potential. Both compounds significantly reduced cell viability in a dose- and time-dependent manner, with slightly higher activity observed for (+)-UA. The A375 melanoma cell line was the most sensitive to (+)-UA, whereas the effect observed in HTB-140 cells was markedly weaker. Both enantiomers exhibited moderate cytotoxicity against WM793 cells, which remained completely resistant to doxorubicin (an anthracycline chemotherapy drug used to treat various cancers) treatment (IC_50_ > 100 µg/mL). Additionally, the combination of UA enantiomers with doxorubicin was investigated. In the A375 and WM793 cell lines, a pronounced synergistic effect was observed for both enantiomers. Particularly high efficacy was noted for the combination with (−)-UA, especially after 24 h of incubation. More complex interactions were observed in the HTB-140 cells, where (+)-UA exhibited strong antagonism, which gradually shifted toward an additive effect over time. In contrast, the effect of (−)-UA was time-dependent—the initial synergism observed after 24 h diminished and eventually transformed into antagonism after 48 h. Moreover, the effect of both enantiomers on cell proliferation was evaluated. The strongest antiproliferative effect was observed in the HTB-140 and WM793 cells, while A375 cells, despite their high sensitivity to the cytotoxic effect, exhibited marked resistance under all tested conditions. Both enantiomers of UA also strongly inhibited the migration of all examined melanoma cell lines, at a low subcytotoxic concentration of 10 µg/mL, with (+)-UA revealing a stronger effect for HTB-140 and A375 cells. In WM793 cells, both compounds acted with similar intensity, and no statistically significant differences were observed [18].

In another study, the impact of UA on viability, proliferation, and gene expression in A-375 human melanoma cells and human epidermal melanocytes was determined. The compound effectively inhibited cancer cell proliferation, with no toxicity to normal cells. Mechanistic studies indicated the induction of apoptosis through the simultaneous activation of the extrinsic and intrinsic pathways. Molecular analysis confirmed an increase in the expression of 61 pro-apoptotic genes (including BNIP3, BCL10, and TP53) and a decrease in the activity of 23 anti-apoptotic genes, including key cell death inhibitors such as BIRC5 (survivin). The study also showed a significant increase in the enzymatic activity of caspase-3 (1.75-fold) and caspase-9 (1.3-fold). Additionally, UA was observed to inhibit the expression of the mutated BRAF (V600E) gene, which is responsible for the uncontrolled increase in melanoma progression [19].

Mariraj et al. investigated the effect of UA on human malignant melanoma G361 cancer cells. Cell viability decreased in a dose- and time-dependent manner following the administration of UA. The test compound also induced apoptosis in the cells, as confirmed by DAPI staining observed under a fluorescence microscope [20].

Some inconsistencies in the observed cytotoxic results for UA, presented as IC_50_ values, can also be noted. These may result from the differences in the cellular assay type used in the studies, the purity of the UA enantiomer, or even from the variabilities in the reagents used for the assays. All these may influence the final effect, as it is especially observed for A375 cells, with the IC_50_ values differing from 20 to even 87.85 µM (Table 1).

**Table 1:** Summary of the anti-melanoma activity of lichen extracts and UA in in vitro models.

Substance Tested	Cell Line	Parameters/IC_50_	Control	Main Findings	Ref.
LICHEN EXTRACTS					
*Cladonia mitis* acetone extract	melanoma: HTB140, A375, WM793; keratinocytes: HaCaT	dose-dependent cytotoxicity; highest potency against A375 cells (IC_50_ range: 41.14–55.73 µg/mL	doxorubicin (reference, IC_50_ = 2.93 µM for A375); Kojic acid (antityrosinase)	No correlation between UA content and extract activity; rather low antityrosinase activity.	[21]
*Usnea* *aurantiaco-atra* extracts (hexane, methanol, DCM)	melanoma: A375	hexane extract: IC_50_ = 5.73 ± 1.19 µg/mL; dichloromethane extract: IC_50_ = 27.02 ± 1.21 µg/mL; methanolic extract: IC_50_ = 293.3 ± 1.17 µg/mL; fraction 4 of hexane extract demonstrated the highest activity (IC_50_ = 0.54 ± 1.05 µg/mL)	BHT (antioxidant control, IC_50_ = 45.65 ± 16.79 µM), untreated cells	UA content influenced IC_50_, but pure commercial UA was 10-fold less active than the extract (IC_50_ = 151.54 ± 2.99 µM) (complex synergy with terpenes/sterols).	[22]
USNIC ACID					
(+)-UA	melanoma: HTB140, A375, WM793; Keratinocytes: HaCaT	Strongest activity against HTB140 cells (IC_50_ = 40.60 µM); A375 cells were the most resistant (IC_50_ = 87.85 µM)	doxorubicin (IC_50_ = 6.94 µM for HTB140 and 2.93 µM for A375); Kojic acid	Anti-melanoma profile distinctly differed from that of the whole *Cladonia mitis* extract.	[21]
(+)-UA	melanoma: UACC-62, B16-F10; fibroblasts: 3T3	UA GI_50_ = 91.48 µM against UACC-62 cells and 138.53 µM against B16-F10 cells; for normal 3T3 fibroblasts, GI_50_ = 120.24 µM	doxorubicin (GI_50_: 0.86 µM for UACC-62 and 0.46 µM for B16-F10)	Moderate anti-melanoma activity; revealed only limited/low toxicity to normal 3T3 fibroblasts.	[16]
UA (isolated from *C. arbuscula*, enantiomer not specified)	melanoma: HTB140; fibroblasts: HSF	EC_50_ = 64.18 µM (24 h, HTB-140 melanoma) EC_50_ = 39.79 µM (72 h, HTB-140 melanoma) For normal HSFs: EC_50_ = 56.05 µM	DMSO (solvent control), untreated cells	Pronounced anti-invasive effect; decreased cell trajectory by ~55%; induced rearrangement of actin cytoskeleton.	[17]
Enantiomers: (+)-UA and (−)-UA	melanoma: HTB140, A375, WM793	For (+)-UA (48 h): IC_50_ = 42.75 µM (HTB140), 34.39 µM (A375) and 87.27 µM (WM793) For (−)-UA (48 h): IC_50_ = 59.89 µM (HTB140), 64.30 µM (A375) and 151.28 µM (WM793)	doxorubicin alone (IC_50_ after 48 h: 3.7 µM for HTB-140, 1.01 µM for A375, and >183.99 µM for WM793); DMSO solvent control.	Slight predominance of (+)-UA toxicity; strong synergism between (−)-UA and doxorubicin to overcome drug resistance in WM793.	[18]
UA (isolated from *Usnea baileyi* mycobiont culture, enantiomer not specified)	melanoma: G361	IC_50_ = 700.80 ± 3.63 µM (with 24 h incubation)	untreated melanoma cells (negative control in MTT assay), DMSO (solvent control); Chloramphenicol (30 µg as positive antibiotic control) and acetone (negative disk control) in antimicrobial assay.	Effectively induced apoptosis in cancer cells, as confirmed by fluorescent DAPI staining.	[20]
(+)-UA	melanoma: A375; human melanocytes	IC_50_ = 20 µM determined at 45 h on A-375 cells; no cytotoxic effect on human epidermal melanocytes up to 25 µM	melanocytes in 0.05% DMSO (vehicle control)	Induced apoptosis via simultaneous activation of extrinsic and intrinsic pathways; ↑ expression of 61 pro-apoptotic genes; ↓ mutated BRAF (*V600E*) expression.	[19]

A375: human melanoma cell line; B16-F10: murine melanoma cell line; G361: human melanoma cell line; HSF: human skin fibroblasts; HTB140: human melanoma cell line; UA: usnic acid; UACC-62: human melanoma cell line; WM793: human melanoma cell line; 3T3: murine fibroblast cell line; ↑ increase; ↓ decrease; further abbreviations are explained in the Abbreviations section.

### 3.2. Wound Healing Potential of Usnic Acid and Its Derivatives

Wound healing potential is another interesting aspect of UA’s impact on skin cells (Figure 3). The effect was evaluated mostly in vitro, but some in vivo experiments were also described. Moreover, a few studies reported the effect of UA derivatives. No information was found on the wound healing effect for (−)-UA.

Wound healing effects of (+)-UA were determined in the scratch wound test, based on the HaCaT cells model, with platelet lysate as a reference point. At a very low concentration (2 µM), the compound significantly enhanced cellular migration and wound healing. Interestingly, the combination of (+)-UA and another lichen metabolite, gyrophoric acid, induced an about fourfold increase in the HaCaT wound closure rate. This combined effect reached an activity comparable to that of the platelet lysate used as a positive control. A combination of lichen compounds can therefore act synergistically, suggesting that crude lichen extracts might reach levels of activity comparable to those obtained with clinically relevant preparations [15].

The wound healing effect was also further described in vivo, where the ability of UA to inhibit hypertrophic scar formation was evaluated in a rabbit ear model. Three weeks after wounding, the animals received intradermal treatment with 2 mg of UA dissolved in 50 μL of DMSO per wound (treatment group, n = 6), 50 μL of DMSO (control group, n = 6), or 50 μL of triamcinolone acetonide acetate (reference group, n = 6), once a week, for a total of four times. The use of UA and triamcinolone improved the quality of the regenerated tissue, which became flatter, softer, and more similar in color to healthy skin, as compared to the control group. Although UA effectively reduced tissue hyperplasia, the effect was weaker than that of triamcinolone. Scars in the UA group retained a more intense color, while those in the steroid group were pale pink and almost flush with the surrounding skin. Masson’s staining revealed significant differences in collagen structure between the study groups. The control group was dominated by thick, densely packed fiber bundles with a chaotic, swirling pattern, while the treatment groups (UA and triamcinolone) showed marked matrix reorganization. UA also effectively inhibited angiogenesis in hypertrophic scar tissues over the 35-day treatment period. Analyses show that UA effectively limits the development of hypertrophic scars in a rabbit model. The mechanism of this effect is closely related to the antiangiogenic properties of the substance, which inhibits endothelial cell migration and their ability to form new vascular structures (tubules). Although the authors did not specify the type of enantiomer used, it was rather (+)-UA, as it was purchased from Sigma-Aldrich (Merck, Darmstadt, Germany) [23].

**Figure 4:**
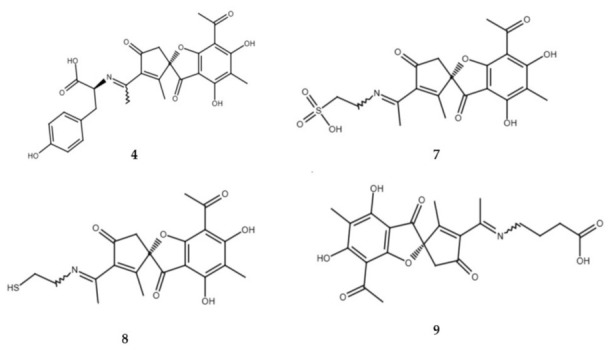
Chemical structures of active enamine derivatives of (+)-UA discussed in the text: L-tyrosine derivative (**4**), taurine derivative (**7**), cysteamine derivative (**8**), and γ-aminobutyric acid (GABA) derivative (**9**). Adapted from [24].

Apart from UA alone, its derivatives were also evaluated. Bruno et al. evaluated the wound healing potential of (+)-UA enamines, using a scratch assay on human skin keratinocytes HaCaT. Compounds **4**, **7**, **8**, and **9** were superior to UA in tissue regeneration (**4**, **7**, and **8** being L-tyrosine, taurine, and cysteamine derivatives, respectively), with the GABA-UA derivative (compound **9**) being the most potent (Figure 4). Its efficacy was almost equal to that of platelet lysate, used as a reference standard. Further in vivo experiments on male rats and albino mice (with 7 animals per experimental group) with incisional and excisional wound models were also performed. In rats, two incisions were made along the spine and sutured, and the animals were treated for 9 days with UA, reference drug Madecassol (containing 1% *Centella asiatica* extract), or the ointment base was applied. In mice, a circular open wound was created; its closure was monitored while applying the above-mentioned preparations. Again, the best results were obtained for compound **9**, which increased skin tensile strength by 47.6% and achieved ~83% wound closure by day 10. Healing activity depended on the presence of acidic groups, and their absence eliminated the effect (e.g., compound **11**, a non-acidic enaminousnic acid derivative used as a negative reference). Compound **8** additionally supported regeneration due to the presence of a thiol group [24].

**Figure 5:**
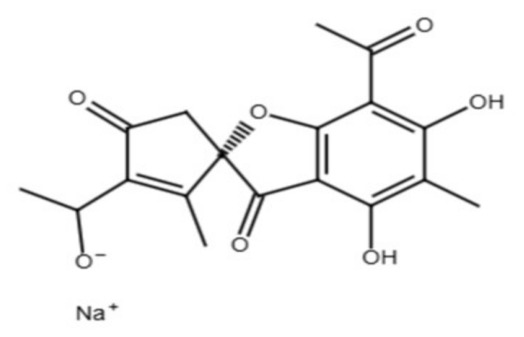
Chemical structure of sodium usnic acid (SUA). Adapted from [25].

In another experiment, the efficacy of topically applied UA sodium salt (Figure 5) at a concentration of 38,400 μg/L on macroscopic and microscopic changes following skin trauma in male rats (64 animals divided into 4 groups, 16 per group) was evaluated. A full-thickness wound model was used in the study, with two circular incisions made. The effects of the compound were compared with an antibiotic (gentamicin) and control groups (no treatment and DMSO solvent). The compounds were applied topically once daily to each wound in a volume of 50 μL for 21 days. The study showed that both UA and gentamicin-treated groups had significantly faster wound healing rates compared to the control groups, which received no treatment. After 14 days of injury, the wounds in the first two groups had completely healed, whereas in the control groups, the healing process was incomplete. UA sodium salt induced a very early release of vascular endothelial growth factor (VEGF) (as early as the first day, while in the control group, it only occurred on the third day), which accelerated the formation of new blood vessels and wound nourishment. VEGF is involved in the inflammatory and proliferation phases of healing, and a steady increase in its levels is characteristic of the early stage of inflammation. This suggests that the tested UA derivative shortened the inflammatory phase, which simultaneously allowed the tissues to more quickly transition to the intensive regeneration stage. Histological analysis confirmed improved collagen fiber alignment, faster epithelialization, and fibroblast growth. The results of UA sodium salt treatment were comparable to those obtained with gentamicin, making it a promising natural therapeutic agent [25].

### 3.3. Photoprotective Activity of Usnic Acid

UA reveals UV-absorbing properties and plays an important role in protecting lichens from the damaging effects of excessive UV light exposure [20]. This unique attribute of UA has also been studied in terms of its photoprotective activity directed to the skin. No information was found for the photoprotective effect of (−)-UA.

In one of the experiments, the photoprotective properties of UA were investigated in vitro by measuring SPF (Sun Protection Factor, which measures efficacy against UVB), PF-UVA (UVA Protection Factor, indicating protection against UVA), and λ_c_ (critical wavelength, an indicator of the filter capacity to protect in the UVA range), but also SUI (Spectral Uniformity Index) and ISP (Ideal Spectral Profile) to determine radiation protection across the entire ultraviolet spectrum. The results for UA allow it to be classified as a UVB photoprotector (SPF = 3.9; PF-UVA = 1.8; λ_c_ = 364 nm; ISP = 53% and SUI = 1.8). For comparison, the positive control ethylhexyl methoxycinnamate (OMC) exhibited values of SPF = 10.4; PF-UVA = 1.6; λc = 330 nm; ISP = 105% and SUI = 1.1. Additionally, the safety profile was assessed using the Photo-Irritancy Factor (PIF) in UV-irradiated human HaCaT keratinocytes. The calculated PIF value for UA was low, as compared to the phototoxic positive control, chlorpromazine (0.7 vs. 5.5, respectively), indicating no phototoxic effect [26]. Another interesting experiment assessed the effect of (+)-UA and its five photodegraded derivatives on UV-induced melanogenesis in epidermal melanocytes. Based on double fluorescence staining analysis using Hoechst 33342/propidium iodide dyes and quantified via a computer-linked fluorescence microscope equipped with the CellSense Dimension system, compounds **2**–**5** demonstrated significant UVA/UVB radiation protection in the tested cells. Tyrosinase expression in epidermal melanocytes exposed to UVB radiation was also calculated compared to the control group, i.e., unexposed to light. All compounds at a dose of 50 μM did not cause a change in tyrosinase activity compared to the control group. Only compound **2** at a dose of 100 μM showed a significant difference in tyrosinase expression compared to the unexposed group. The results of this experiment indicate that photodegraded derivatives of (+)-UA exhibit significant UV radiation protection, with significantly lower toxicity to melanocytes than UA [27].

UA enantiomers were compared as candidates for UV absorbers in cosmetic products in terms of their photoprotective properties, skin-penetrating ability, and also the impact on normal human skin cells: keratinocytes, melanocytes, and fibroblasts. Both enantiomers demonstrated high penetration through the skin barrier, regardless of the dose. Low toxicity was observed for both enantiomers, occurring only with longer exposures (after 48 and 72 h) at the highest tested concentration (100 µg/mL), with HaCaT as the most sensitive cells, and slightly higher toxicity of (−)-UA, as compared to the other enantiomer. The photoprotective activity of (+)-UA and the reference octocrylene was very similar, whereas the combination of both substances showed an increase in SPF and PF-UVA values by 25 and 13.25%, respectively, compared to octocrylene alone. Due to its photostability, photoprotection, and good safety profile for skin cells, dextrorotatory UA could potentially be used as a UV filter in cosmetic products [28]. Another experiment investigated the photoprotective effect of UA on irradiated, as compared to the effect on non-irradiated HaCaT cells. The results indicated that UA, which was characterized by a greater photosensitizing capacity, had a more detrimental effect on HaCaT cells, causing a significant decrease in mitochondrial metabolic activity, loss of cell membrane integrity, induction of apoptosis, and disruption of cytoskeleton structure [29]. A similar experiment analyzed the photoprotective properties of UA, measured by the determined SPF, both in vitro and in human volunteers. In the in vitro experiment, human T-lymphoma cell suspensions were irradiated through a thin layer of a light-absorbing substance. OMC was used as the reference substance, 4-tert-butyl-4′-methoxy dibenzoylmethane (BM-DBM) was additionally used as a reference UVA filter, while untreated irradiated cell suspensions without the tested compounds served as controls in the MTT viability assay. The membrane protection factors (MPF) determined in the experiment were higher for UA than for OMC (4.5 and 3.2, respectively). In a subsequent part of the experiment, the effect was also examined in healthy volunteers (n = 5). A solution of UA was applied to a small area of the skin on their back (0.04 mg of UA/cm^2^ of skin surface). Nivea Sun Spray LSF 5 was used as the control substance. The calculated UVPF values ranged from 2.0 to 4.2. The mean UVPF value for Nivea was 4.2, while UA achieved a value of 4.1 [30].

### 3.4. Novel Topical Formulations of Usnic Acid to Enhance the Efficacy

**Figure 6:**
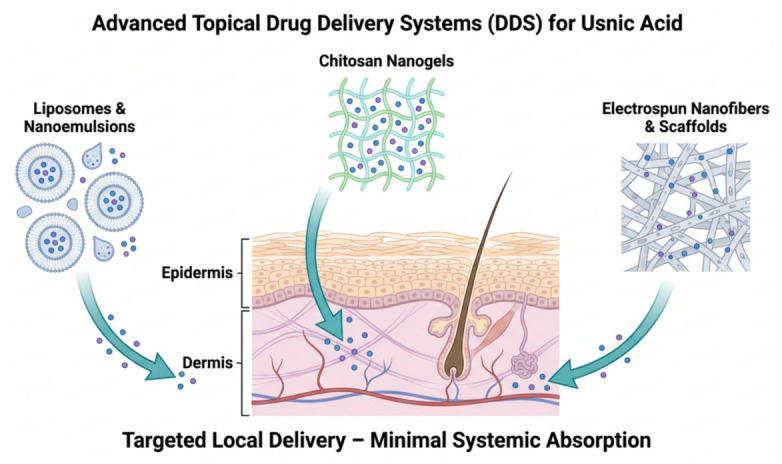
Overview of advanced topical drug delivery systems (DDS) for localized UA administration. Nanostructured carriers, including liposomes, nanoemulsions, chitosan nanogels, and electrospun fibrous scaffolds, enhance skin permeability and ensure targeted delivery into the epidermal and dermal layers while minimizing systemic absorption and associated hepatotoxicity.

Although UA is generally lipophilic and can easily penetrate skin layers, some efforts have been made to enhance its effectiveness by the development of novel formulations for the potential topical use (Table 2, Figure 6). One of the examples was an in vitro evaluation of a novel treatment approach for cutaneous leishmaniasis, based on gelatin membranes containing liposome-encapsulated UA (UAL). UA concentration of 0.8 µg/mL was shown to significantly and irreversibly reduce the viability of *Leishmania braziliensis* parasites. UA-containing membranes had no toxic effects toward human macrophages or fibroblasts, used as control groups, suggesting their safety for application to healthy tissues. The developed formulation not only effectively reduced parasite numbers but also supported the regeneration of damaged tissues, demonstrating the characteristics of a promising therapeutic agent for the treatment of local symptoms of *Leishmania braziliensis* infection [31].

In another experiment, a nanoemulsion combining UA with cinnamon oil (CUN) was developed to investigate whether this nanoparticle-based formulation could inhibit skin tumor development, induced by topical application of DMBA and croton oil, in Swiss albino mice. The animals (10 mice per group) received topical application of acetone (negative control group), DMBA, and croton oil without treatment (positive carcinogenic control group) or the developed nanoemulsion. A formulation containing a conventional blend of cinnamon oil and UA (CUB) was also used as a comparative treatment group. The obtained results indicate synergistic effects of cinnamon oil and UA, observed as the decreased number of tumors (papillomas) and their size as compared to the positive control group, with CUN demonstrating significantly higher efficacy than CUB. The preparation restored the natural levels of antioxidant enzymes in the skin (such as catalase and superoxide dismutase), which are destroyed by the carcinogenic process. Histopathological studies showed reduced inflammatory cell infiltration and less tissue damage in the CUN group [32].

Another experiment focused on the potential use of UA nanogels in the treatment of oral ulcers. Nanogels are innovative drug carriers that, thanks to their microscopic structure and ability to swell, are suitable for local therapy in the oral cavity. They enable more effective dissolution of therapeutic substances and their gradual release, reducing the need for frequent application. The nanogels were obtained by ionotropic gelation, exploiting electrostatic interactions between cationic chitosan (CS) and polyanionic cross-linking agents: sodium tripolyphosphate (TPP; formulations F22–F33) and bovine serum albumin (BSA; formulations F1–F21). Based on the results, five formulations were selected as optimal and additionally enriched with usnic acid: F4, F10, F13, F28, and F31. Analysis of UA content revealed a high percentage of UA in the nanogels (91 to 96.6%). The tested formulations demonstrated varying active substance release dynamics depending on their composition. Systems based on CS and BSA (F4, F10, F13) were characterized by a similar release profile, reaching nearly 90% of the dose after 24 h. In the initial phase (first hour), a release of 15–20% was observed, which is within the standards for this type of carrier. In contrast, nanogels F28 and F31 released the drug more uniformly, with a release rate of only 30% after 24 h, likely due to the high viscosity of these formulations. Analysis of microbiological activity demonstrated that free UA exhibited MIC (minimum inhibitory concentration) values against *B. cereus* and *S. aureus* strains of 0.25 mg/mL and 1 mg/mL, respectively. For Gram-negative bacteria such as *E. coli* and *P. aeruginosa*, this parameter remained at 3 mg/mL. A 25% DMSO solution served as the solvent control in antimicrobial assays. In the subsequent agar well diffusion assay against *B. cereus*, the optimized nanogel formulations (F10 and F13) produced distinct zone inhibition diameters (19 mm and 21 mm, respectively), confirming that the encapsulated UA retains its high antimicrobial potential within the polymeric matrix. These findings underscore the strong therapeutic potential of the developed formulations, predisposing them to potential use in tissue regeneration and the treatment of infected mucosal wounds. Bovine buccal mucosa was used for subsequent ex vivo permeation and penetration studies for formulations F10 and F13. The F10 formulation demonstrated a permeation rate of 1.068% and a penetration rate of 1.392% after 24 h. For the F13 formulation, these values were even lower: 0.386% and 0.868%. Analysis of mucoadhesive parameters determined that F10 and F13 had values of 0.193 and 0.215, respectively. The obtained data indicated a higher adhesive potential for the F13 formulation, which, combined with the favorable in vitro release profile and ex vivo results, determined the selection of this particular formulation for further testing. Finally, an in vivo experiment was performed in male rabbits (n = 21), with circular ulcers of the labial tissue of the oral cavity induced by acetic acid. The animals were divided into groups treated for 12 days with blank nanogel formulations without UA, UA-loaded F13 nanogel, or sterile saline solution serving as the control condition. In the case of the F13 formulation with added UA, an increased angiogenic process was observed compared to the other study groups. Increased fibroblast proliferation, faster collagen fiber synthesis, and more abundant vascularization of the ulcerated area were observed. The study conclusions confirm that UA contained in the F13 nanogel acts as the primary factor stimulating healing, initiating a cascade of biological processes leading to complete mucosal reconstruction [33].

Another study aimed to evaluate the effect of collagen films containing liposome-encapsulated UA on the healing of skin burns in rats. The experiment was conducted on a group of 45 Wistar rats with second-degree burns. The animals were assigned to nine groups, receiving pure collagen films (COL), collagen films with empty liposomes (PHO), or collagen films with UAL. Observations were conducted at three time points—7, 14, and 21 days after the start of treatment. In the UAL group, day 7 showed intense, widespread neutrophil infiltration, whereas control groups exhibited milder, edge-limited inflammation, indicating faster immune activation. By day 14, inflammation in UAL decreased to moderate with plasma cells present, while remaining strong in controls. By day 21, inflammation in UAL had nearly resolved, suggesting accelerated healing. Epithelialization (ER%) was significantly higher in UAL at day 7, with no differences at later stages, likely due to early release of UA during collagen matrix absorption. UAL also exhibited an earlier transition from type III to type I collagen (day 14). By day 21, scars resembled normal dermis, unlike controls, where excess type I collagen increased hypertrophic scar risk. UA promoted balanced collagen deposition and early fibroblast–myofibroblast transition. By day 21, myofibroblasts decreased in UAL, indicating normal healing completion, while persistence in controls suggested delayed regeneration [13].

The effect of a gelatin membrane containing liposomes with (+)-UA on the healing of dermal burns was assessed in a porcine model. Due to the poor solubility of the test compound in an aqueous environment, it was encapsulated in liposomes, which, thanks to their amphiphilic nature, effectively transport hydrophobic substances. The use of liposomal systems allows for controlled release of the active substance, resulting in higher bioavailability and reduced toxic effects. Nine male pigs underwent three dorsal burns and were randomly treated with silver sulfadiazine ointment (SDZ), DuoDerme1 wound dressing (GDU), or a gelatin membrane containing UAL every four days for 8, 18, and 30 days. UA was released from UAL rapidly for the first four hours, and then continued to deliver it in a steady and controlled manner throughout the day, achieving 98.15% (41.37 µg cm^−2^) of released UA. Transdermal absorption studies of UA from UAL were performed by comparing the detected amounts of UA (µg cm^−2^) in the epidermis, dermis, and receptor fluid. The amount of UA in the epidermis was 3.54 ± 0.79 µg cm^−2^, while in the dermis it was 13.64 ± 0.17 µg cm^−2^. The results show that the active substance tended to accumulate in the skin in the dermis in larger amounts compared to the epidermis and was likely able to be absorbed systemically. Receptor fluid, which is considered the amount absorbed into the systemic circulation, was measured after 24 h and was 4.1 ± 0.32 µg cm^−2^. No clinical signs of purulent exudate, cellulitis, or hypertrophic or atrophic scarring were observed, suggesting that healing proceeded uneventfully. The mean burn wound area gradually decreased during burn healing in all treatment groups, with the greatest reduction observed between days 8 and 18. However, no significant differences were observed between the mean burn areas of the study groups. Gelatin membrane with UAL significantly affected the quality of newly formed granulation tissue, by accelerating granulation tissue maturation, increasing collagen fiber density and organization, supporting proper vascularization, and helping transition the wound from the inflammatory to the repair phase, resulting in the formation of tissue more similar to healthy skin. The developed drug delivery system in the form of a gelatin film likely has potential for clinical use in the treatment of burn wounds, thanks to its ability to control the release of UA and stimulate effective regeneration of damaged tissues [34].

UA is especially known for its antibacterial and antibiofilm properties, including the pathogens causing skin infections, which were recently reviewed in detail by Li et al. [35]. Some studies also focused on the novel formulations of UA in terms of its antibacterial effect. One of such experiments aimed to design and characterize an electrospun nanofibrous mesh composed of polyvinyl alcohol (PVA), chitosan (CS), and UA for use in wound healing as a modern dressing supporting tissue regeneration. In vitro studies assessed biocompatibility (XTT test, GSH test, and fluorescence microscopy) and antimicrobial properties against *Staphylococcus aureus*. Untreated fibroblasts cultured without the tested meshes served as the control group in cytocompatibility assays, whereas meshes without UA were used as formulation controls for comparison of antibacterial activity and biofilm inhibition. Cell viability analysis confirmed the high biocompatibility of both tested materials: 5% PVA with 2% CS and 5% PVA with 2% CS with added UA, with cell survival exceeding 90%. The addition of UA stimulated cell proliferation, increasing their viability by nearly 30% compared to the control group after 72 h. The GSH test confirmed that the materials did not cause oxidative stress and, therefore, premature cell apoptosis. The addition of UA endowed the meshes with antibacterial properties. A significant inhibition of *Staphylococcus aureus* biofilm development was observed compared to the control group, with this effect lasting up to 72 h. The material was characterized by its ability to swell in physiological fluids (SBF, PBS), which promotes the absorption of wound exudate. The combination of the mechanical advantages of PVA, the biological activity of chitosan, and the therapeutic potential of UA makes the obtained nanostructures a promising alternative to traditional methods of treating burn and chronic wounds [36].

A similar study developed and optimized a functional medical dressing with a highly ordered microfiber structure, combining the regenerative properties of collagen with the antibacterial action of UA against *E. coli* and *S. thermophilus*, using polycaprolactone (PCL) as the structural framework. Its mechanical strength and near-field electrospinning (NFES) processability provided the dressing with a stable, ordered form and the durability necessary for tissue regeneration. Genipin, was used to cross-link the fibers. Pure PCL fibers and PCL/collagen fibers without UA served as reference materials for comparison of antibacterial activity, physicochemical properties, and cellular responses. Untreated bacterial cultures and HUVEC cells cultured without the tested materials were used as control groups in microbiological and biocompatibility assays. The dressing containing 1% UA demonstrated a strong inhibitory effect on the growth of both bacterial strains. Cross-linking with genipin resulted in improved mechanical and tensile strength of the composite fibers (AC-PCUCF). Creating a more compact collagen structure allowed for slower and prolonged release of UA (up to 72 h), ensuring long-term wound protection and, thus, being beneficial for the healing process. The addition of 20% collagen provided the composite fiber improved hydrophilicity and water absorption (reaching up to 500%), which is also crucial for wound therapy. Furthermore, 20% collagen significantly accelerated the adhesion and proliferation of HUVEC cells, compared to pure polycaprolactone. The results of this study indicate that this material may be a promising solution in tissue engineering [37]. Similarly, the development process and properties of a modern wound dressing that combines polyurethane foam (PU), the polymer polyaniline (PANI), and UA were described. A layer of polyaniline was deposited on a polyurethane carrier using in situ polymerization. This resulted in a porous material, into which UA was then incorporated. Antibacterial activity was assessed against *Escherichia coli* and *Staphylococcus aureus*. The results demonstrated a 75% inhibition of biofilm formation (polyaniline alone inhibited this by approximately 20%). Bacterial adhesion to the foam was also reduced by 94% for *E. coli* and 90.22% for *S. aureus*. These results support the potential future use of this wound dressing [38].

Zha et al. described a functionalized tissue scaffold based on silk fibroin (SF) doped with UA, which was intended to have antibacterial and pro-regenerative functions. The material was produced by electrospinning, obtaining a composite (USCS) with a homogeneous nanofiber structure. Mechanical analysis of USCS scaffolds showed that elasticity depended mainly on silk fibroin (SF) concentration, not β-sheet content. Increasing SF from 18% to 20% raised elongation at break (0.92% → 2.52%). Methanol treatment enhanced tensile strength (up to 2.61 MPa), while 0.5% UA had no effect on strength. Protease XIV studies confirmed that higher SF content and β-sheet formation improved stability, extending degradation time to 14 days after methanol treatment. This allowed for the increased scaffold durability to tissue regeneration while maintaining an appropriate water vapor transmission rate. UA release depended on scaffold structure: untreated samples showed rapid release (>58% on day 1), whereas methanol treatment reduced initial release (~32%) and prolonged it to 10 days via β-sheet–induced diffusion barriers. USCS exhibited strong antimicrobial activity, particularly against Gram-positive bacteria (*S. aureus*, *S. pyogenes*), with controlled UA release. CCK-8 assays confirmed biocompatibility. In a subsequent full-thickness skin wound model in mice, USCS showed superior healing compared to controls and 3M Tegaderm, achieving >53% closure by day 3 and ~96.5% by day 12. The synergy between SF and sustained UA release was key to enhanced regeneration, supporting its potential for chronic wound treatment. Although silk fibroin itself has pro-regenerative properties, the results suggest that synergy with the sustained release of UA is a key factor in accelerating tissue repair. The proposed strategy of combining SF with UA opens a new path in the treatment of chronic wounds, offering a material with optimal mechanical characteristics and high therapeutic potential [39].

**Table 2:** Advanced topical formulations of UA and their therapeutic efficacy in wound healing and infection models.

Formulation/Source	Composition	Model	Parameters	Main Findings	Ref.
IN VITRO STUDIES					
Gelatin membrane with liposomes (UAL)	gelatin, liposome-encapsulated UA (enantiomer not specified)	(*Leishmania braziliensis*); Human macrophages and fibroblasts	UA concentration: 2.32 µM	Significantly and irreversibly reduced parasite viability; no toxic effect on healthy human tissues; supported tissue regeneration.	[31]
Electrospun Nanofibrous Mesh	polyvinyl alcohol (PVA), chitosan (CS), UA (enantiomer not specified)	cytocompatibility and antimicrobial assays	cell survival > 90%; addition of UA stimulated proliferation by ~30% after 72 h	High swelling capacity in physiological fluids; significant inhibition of *Staphylococcus aureus* biofilm development lasting up to 72 h.	[36]
Composite Biomedical Dressing	polycaprolactone (PCL), collagen (20%), UA (1%; enantiomer not specified), genipin cross-linker	microbiological and cell assays	order microfiber structure (NFES); slowed and prolonged UA release up to 72 h	Strong inhibitory effect against *E. coli* and *S. thermophilus*; improved hydrophilicity (water absorption up to 500%); accelerated HUVEC cell adhesion.	[37]
Porrous Wound Dressing	polyurethane foam (PU), polyaniline (PANI), UA (enantiomer not specified)	bacterial adhesion	PU carrier with in situ polymerized PANI layer	75% inhibition of biofilm formation; reduced bacterial adhesion by 94% for *E. coli* and 90.22% for *S. aureus*.	[38]
Chitosan-BSA Nanogel (F13)	chitosan (CS), Bovine Serum Albumin (BSA), UA (enantiomer not specified)	in vitro, ex vivo (bovine buccal mucosa), in vivo (male rabbits)	high UA content (~95%); released ~90% of the dose after 24 h; ex vivo permeation: 0.386%, penetration: 0.868%	Strong antimicrobial activity (*B. cereus* MIC: 726.07 µM, *S. aureus* MIC: 2904.28 µM); in vivo: stimulated angiogenesis, fibroblast proliferation, and faster collagen synthesis.	[33]
Functionalized Tissue Scaffold	silk fibroin (SF), UA (0.5%; enantiomer not specified), methanol conditioning	in vitro CCK-8 assay, in vivo full-thickness skin wound (mice)	methanol stabilization extended UA diffusion time to 10 days; water vapor transmission rate (WVTR) optimized	Broad spectrum of antimicrobial activity (high sensitivity to Gram-positive bacteria); in vivo wound closure rate over 53% on day 3, reaching 96.5% on day 12 (superior to 3M Tegaderm).	[39]
IN VIVO AND EX VIVO STUDIES					
Nanoemulsion (CNS)	cinnamon oil, UA (enantiomer not specified), Tween 80, ethanol	in vivo skin tumor model (Swiss albino mice)	nanometric droplets with enhanced skin permeability	Synergistic effects; reduced papilloma number and size; restored natural antioxidant enzymes (catalase, SOD); decreased inflammatory cell infiltration.	[32]
Collagen films with liposomes (UAL)	pure collagen films, liposome-encapsulated UA (enantiomer not specified)	in vivo second-degree skin burns (Wistar rats)	evaluations at day 7, 14, and 21; Epithelialization index (ER%)	Accelerated initial immune response (neutrophils at day 7); optimized burn regeneration by promoting rapid transition from type III to type I collagen; prevented hypertrophic scarring.	[13]
Gelatin membrane with liposomes (UAL)	gelatin membrane, liposome-encapsulated (+)-UA	in vivo dermal burns (porcine model)	rapid initial release (38.70% in 4 h), controlled steady release up to 98.15% in 24 h; accumulation in dermis (13.64 µg/cm^2^)	Uneventful healing with no clinical signs of necrosis or hypertrophic scars; accelerated granulation tissue maturation and density.	[34]

See Abbreviations section for details.

### 3.5. Usnic Acid-Rich Lichen Extracts and Their Impact on Normal and Cancer Skin Cells

Apart from the studies reporting topical uses of UA alone, there are also some experiments describing the effects of UA-rich extracts from different lichen species on cancer and normal skin cells (Table 1).

Cytotoxic activity of acetone extracts from *Cladonia mitis* was examined in relation to UA content. An in vitro model consisting of three melanoma cell lines: HTB-140, A375, WM793, and normal skin keratinocytes, HaCaT, was used as selectivity indicators. An antityrosinase assay was also performed to further assess the antimelanoma potential of *Cladonia mitis* extracts. The results demonstrated dose-dependent cytotoxic activity of the tested extracts and UA itself, but no relationship was observed between UA content and the activity of the extracts. The antimelanoma profile of *Cladonia mitis* extracts differed from that of UA, as the compound demonstrated the strongest activity against HTB-140 cells, while A375 cells were the most resistant. However, the cytotoxic effects of the extracts were quite the opposite. A375 melanoma cells were the most sensitive to the tested extracts. Furthermore, the extracts demonstrated varying, but rather low, antityrosinase activity [21]. In another experiment, the cytotoxic and antioxidant activity of hexane, methanol, and dichloromethane extracts from the Antarctic lichen *Usnea aurantiaco-atra* (Jacq) Bory was assessed. The study confirmed that UA is a key compound present in all three extracts, but in varying amounts. The A-375 melanoma cell line was used to determine cytotoxicity. The results revealed that the hexane extract possessed the highest cytotoxicity, followed by the dichloromethane extract, while the methanolic extract showed low or no activity. Fraction 4 of the hexane extract was the only one to contain sterols and a high percentage of linoleic acid, and it was this fraction that demonstrated the highest activity against melanoma A375 cells among all extracts (IC_50_ = 0.54 ± 1.05 µg/mL). The results suggest that the amount of UA in each extract directly influenced the IC_50_ value for the melanoma cell line. When commercial UA was added at the same concentration as in the extracts and under the same conditions, 10-fold higher IC_50_ values were observed (52.18 ± 1.03 µg/mL) than those obtained with the extract [22].

In another experiment, the biological properties of acetone extracts from *Evernia prunastri* were analyzed. HPLC–UV chromatographic analysis enabled the identification of key compounds in *Evernia prunastri*: UA, atranorin, chloroatranorin, physodic acid, and evernoic acid. Cytotoxic activity was determined against the human FemX melanoma cell line. The acetone extract of *E. prunastri* exhibited moderate cytotoxic activity against the tested FemX melanoma cells. Additionally, DNA content analysis using flow cytometry determined the effect of the tested samples on the cell cycle in FemX cells. An increase in the number of cells containing DNA below the G1 phase was observed, which may indicate a pro-apoptotic effect of the tested compounds. However, the percentage of cells in the S phase and G2/M phase was significantly lower compared to the control group. The results of this study confirm the anticancer activity of lichen extracts and their compounds in vitro [40].

## 4. Conclusions and Future Perspectives

UA remains one of the most promising lichen-derived secondary metabolites, demonstrating a unique dual-action profile in dermatological applications. On one hand, UA exhibits profound anti-melanoma activity in vitro, while on the other hand, in non-cancerous models, UA significantly accelerates wound healing and tissue regeneration. However, there are still some limitations of the studies cited in the review. First, most of the studies focused mainly on the in vitro approach, which makes their results only preliminary. Second, almost no human trials exist, with the exception of a small study on the photoprotective effect of UA in healthy volunteers and the reports on its allergic potential. Moreover, it should be underlined that the data on enantiomeric-specific safety of UA is really scarce and their results are ambiguous, as some in vitro studies indicate higher safety of (+)-UA, while the case studies on allergic potential suggest that the enantiomer is more sensitizing than (−)-UA. All these needs further in-depth studies, comparing the effectiveness and safety of both enantiomers.

Although UA is used in some cosmetic products, a number of clinical translation barriers still exist for its medicinal use. These include the stated hepatotoxicity upon oral administration, poor solubility in water, scarce data on its bioavailability (especially in terms of skin penetration), and a lack of results of human trials to support the potential use, or some regulatory hurdles. As highlighted in the recent literature, topical nanocarriers restrict the action of UA to the target dermal layers, thereby mitigating the risk of systemic absorption and hepatotoxic side effects, but there are still a lot of animal and human trials needed to clearly indicate the effectiveness and safety of UA.

Therefore, further studies should focus on the following key areas: (i) in vivo safety profiles: conducting rigorous, long-term in vivo toxicity studies in higher animal models to establish precise safe dosage thresholds for chronic topical application; (ii) synergistic formulations: investigating the molecular synergy between UA enantiomers and conventional chemotherapeutic agents (e.g., doxorubicin) within smart nano-vehicles to overcome multi-drug resistance in advanced melanoma; (iii) standardization of extracts: evaluating whether standardized, UA-rich lichen extracts provide superior therapeutic efficacy over pure isolated enantiomers due to potential synergistic effects with co-existing lichen terpenoids and sterols.

## Data Availability

No new data were created within the article.

## Abbreviations

The following abbreviations are used in this manuscript:

3T3	Murine embryonic fibroblast cell line
A375	Human malignant melanoma cell line
AC-PCUCF	Crosslinked polycaprolactone/collagen/usnic acid composite fibers (after crosslinking PCUCF)
B16-F10	Murine melanoma cell line
BHT	Butylated hydroxytoluene
BRAF	B-Raf proto-oncogene, serine/threonine kinase
BSA	Bovine Serum Albumin
CCK-8	Cell Counting Kit-8
CUB	Conventional blend of cinnamon oil and usnic acid
CS	Chitosan
DAPI	4′,6-diamidino-2-phenylindole
DDS	Drug Delivery System
DMBA	7,12-dimethylbenz(a)anthracene
DMSO	Dimethyl sulfoxide
DNA	Deoxyribonucleic acid
FemX	Human melanoma cell line
EC_50_	Half maximal effective concentration
ER%	Epithelialization index
G361	Human melanoma skin cancer cell line
GI_50_	Half maximal growth inhibition concentration
HaCaT	Normal human skin keratinocytes
HPLC–UV	High-performance liquid chromatography coupled with ultraviolet detection
HSF	Human skin fibroblasts
HTB-140	Human melanoma cells derived from a metastatic site
HUVEC	Human umbilical vein endothelial cells
IC_50_	Half maximal inhibitory concentration
ISP	Ideal Spectral Profile
MIC	Minimum inhibitory concentration
NFES	Near-field electrospinning
PANI	Polyaniline
PBS	Phosphate-buffered saline
PCL	Polycaprolactone
PIF	Photo-Irritancy Factor
PU	Polyurethane
PVA	Polyvinyl alcohol
SBF	Simulated body fluid
SF	Silk fibroin
SOD	Superoxide dismutase
SPF	Sun Protection Factor
SDZ	Silver sulfadiazine ointment
SUI	Spectral Uniformity Index
UA	Usnic acid
UACC-62	Human melanoma cell line
UAL	Liposome-encapsulated usnic acid
USCS	Usnic acid-functionalized silk fibroin composite scaffolds
VEGF	Vascular endothelial growth factor
WM793	Stage I primary human melanoma cell line
WVTR	Water vapor transmission rate
XTT	2,3-bis-(2-methoxy-4-nitro-5-sulfophenyl)-2H-tetrazolium-5-carboxanilide (cell viability assay)
λc	Critical wave length
